# Transmission of Schmallenberg virus in a housed dairy herd in the UK

**DOI:** 10.1136/vr.101983

**Published:** 2013-11-06

**Authors:** A. E. Shaw, D. J. Mellor, B. V. Purse, P. E. Shaw, B. F. McCorkell, M. Palmarini

**Affiliations:** 1 MRC-University of Glasgow Centre for Virus Research, Institute of Infection, Immunity and Inflammation, College of Medical, Veterinary and Life Sciences, University of Glasgow, Glasgow G61 1QH, UK; 2 Scottish Centre for Production Animal Health and Food Safety, Large Animal Clinical Sciences and Public Health, School of Veterinary Medicine, College of Medical, Veterinary and Life Sciences, University of Glasgow, Glasgow G61 1QH, UK; 3 Centre for Ecology & Hydrology, Bush Estate, Penicuik, Midlothian EH26 0QB, UK; 4 Bishops Farm, Bushton, Swindon, Wiltshire, UK; 5 The George Veterinary Group, High Street, Malmesbury, Wiltshire, UK

**Keywords:** Arthropod-borne infections (arboviruses), Culicoides, Schmallenberg virus, Epidemiology

Schmallenberg virus (SBV) is a recently emerged *Orthobunyavirus* of ruminants originally discovered in 2011 near the town of Schmallenberg in Germany (Hoffmann and others 2012). SBV appears to have entered Europe in the summer of 2011 and has since spread rapidly across much of central and northern Europe. Seroprevalence in some areas has been reported close to 100 per cent (Tarlinton and others 2012, Meroc and others 2013). Viruses closely related to SBV are known to be transmitted by *Culicoides* biting midges (Jennings and Mellor 1989), and field studies have shown the presence of SBV RNA in *Culicoides* species in several affected countries (Rasmussen and others 2012, Elbers and others 2013).

SBV infection is associated with abortion and malformations in cattle and sheep, and has been shown to be neurotropic in lambs and calves infected in utero (van den Brom and others 2012, Varela and others 2013). In dairy cattle, an ‘acute’ form of the disease associated with a drop in milk yield, diarrhoea and mild pyrexia has also been observed. Here we report a within-herd study on a typical dairy farm located in southern England during 2012. The farm runs a dairy herd comprising approximately 230 Holstein cows, approximately 150 of which represent a milking herd. Importantly, no animals were imported onto the farm during the period of this study.

**FIG 1:**
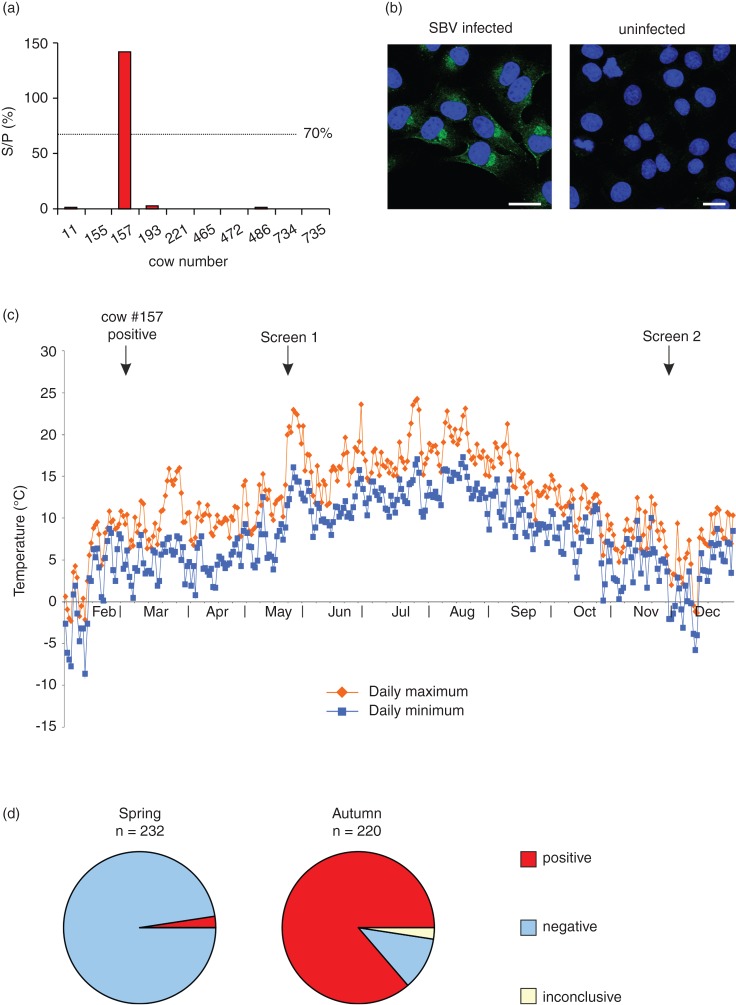
(a) Sera of a selection of cattle tested for the presence of antibodies to Schmallenberg virus (SBV) in February 2012. ­Sample-to-positive ratios (S/P) are expressed as percentages and values >70 per cent are regarded as positive. All animals were negative with the exception of cow #157. (b) SBV infected (left) and uninfected (right) BHK_21_ cells were immunolabelled with sera from cow #157 and analysed by immunofluorescence microscopy. Fluorescent signal was only observed in cells infected with SBV. Bar=10μm. (c) Maximum and minimum daily temperatures for the period February–December 2012 at the nearest weather station (Lyneham, approximately 4.8 km from the farm described in this study), with arrows indicating dates of significance during this study. Data were obtained from the UK Meteorological Office, Met Office Integrated Data Archive System (MIDAS) Land and Marine Surface Stations Data (1853-current), NCAS British Atmospheric Data Centre, 2012. ‘Screen 1′ and ′Screen 2’ represent the points of whole-herd screening for SBV antibodies. (d) Seroprevalence was determined for the entire herd in the spring (May) and autumn (November) 2012, and was found to rise from 1.7 per cent in the spring to 89.1 per cent in the autumn.

In February 2012, a cow (#157) aborted close to term. Suspecting SBV as the cause of abortion, we sampled the affected cow as well as nine additional animals using an indirect SBV antibody ELISA (IDvet). All animals were seronegative for SBV with the exception of cow #157 (Fig 1a). An identical result was obtained upon repeat testing using a second set of blood samples. We further confirmed the presence of anti-SBV antibodies by virus neutralisation assay (Loeffen and others 2012) (data not shown), and immunofluorescence using SBV or mock-infected BHK_21_ cells and sera from cow #157 (Fig 1b). Fluorescent signal was only observed in cells infected with SBV, while no cross-reaction was observed in uninfected cells. Together, these data indicate that SBV infection was present at least as far north as 51.5°N in the UK by February 2012, merely six to eight months after its first recorded appearance in Germany (Hoffmann and others 2012).

Considering arbovirus replication within insect vectors, and the biting activity of the midges required for transmission is inherently reliant upon the ambient temperature, it is interesting to note that in the period immediately prior to the sampling of cow #157, the maximum temperature only reached approximately 10°C (Fig 1c). Using isotype-specific antibody ELISAs, we found IgG but not IgM antibodies in the serum of cow #157 (data not shown). These data suggest cow #157 had been infected for more than 10–14 days prior to sampling, although it is difficult to speculate the exact time of infection.

In May 2012 we screened the entire herd for the presence of SBV antibodies. Seroprevalence among all the animals tested in May was 1.7 per cent (n=232). Subsequently, we retested the herd in November 2012 (towards the end of the midge season), whereupon seroprevalence had risen to 89.1 per cent (n=220, Fig 1e).

During the period between spring and autumn samplings, numerous clinical cases similar to the ‘acute’ form of SBV infection were observed in the herd, with a sudden drop in milk yield for up to a week, followed by recovery, as described in other herds experiencing SBV infection. Similarly, a general increase in diarrhoea was observed among the herd during the summer period, although this observation is difficult to measure, may be multifactorial and retains an element of subjectivity. The dispersed nature of the episodes of acute disease suggests that the spread of infection proceeded over a protracted period of time, although it is difficult to retroactively diagnose acute SBV infection.

Interestingly, the heavy rainfall during the summer period resulted in the milking herd being at pasture on only two occasions (in total four days between May 31 and June 2, and between June 21 and 23) in the whole of 2012. In the remaining time, the herd was housed in open-plan sheds used commonly in the UK, with openings that make them freely accessible to insects.

The study reported here has uncovered valuable insights not necessarily revealed by national serosurveillance screens, and largely concurs with data from other studies of SBV ((EFSA) 2013, Meroc and others 2013, Wernike and others 2013). However, in contrast with some studies, we found a large increase in SBV herd prevalence during a period in which many of the animals were housed (Tarlinton and Daly 2013). For exophilic species of midge, for example, *Culicoides imicola* (Kieffer), stabling during periods of vector activity has historically been used as a way in which to reduce transmission risk (Meiswinkel and others 2007, 2000). This study, as well as previous reports during the recent Bluetongue virus epizootics, reaffirms that housing animals in farm buildings typical of those in the UK during periods of vector activity is not an effective measure against *Culicoides*-borne arbovirus infections in northern Europe, where the predominant *Culicoides* species are those of the *C obsoletus* species complex (Meiswinkel and others 2008, Baylis and others 2010, Viennet and others 2012). Heavy rain has been shown to suppress outdoors rather than indoors *Culicoides* activity (Baylis and others 2010), and it is therefore reasonable to suggest that substantial *Culicoides*-borne SBV transmission occurred inside the sheds during the summer of 2012. Clearly, outbreaks of arboviral diseases can still occur even when animals are not at pasture. It remains possible that significant ‘midge-proofing’ of buildings may offer some protection from midge-borne transmission, although this is likely to additionally depend upon climatic variables, the local landscape and husbandry practices. Further work is required to determine whether such measures are economically viable and the contexts in which they are effective.

Interestingly, the first case (cow #157) identified at this farm was diagnosed in February 2012. Therefore, cow #157 must have been infected either during the winter, when the outside temperature was never above 10°C, or in the summer/autumn of 2011 before or soon after the discovery of SBV in Germany.
